# Exploring the relationship between susceptibility to canine leishmaniosis and anti-*Phlebotomus perniciosus* saliva antibodies in Ibizan hounds and dogs of other breeds in Mallorca, Spain

**DOI:** 10.1186/s13071-020-3992-8

**Published:** 2020-04-21

**Authors:** Alexis C. Burnham, Laura Ordeix, M. Magdalena Alcover, Pamela Martínez-Orellana, Sara Montserrat-Sangrà, Laura Willen, Tatiana Spitzova, Petr Volf, Laia Solano-Gallego

**Affiliations:** 1 Departament de Medicina i Cirurgia Animals, Facultat de Veterinària, Universitat Autònoma de Barcelona, Bellaterra, Spain; 2 Servei de Dermatologia, Fundació Hospital Clínic Veterinari, Universitat Autònoma de Barcelona, Bellaterra, Spain; 3 Departament de Biologia, Sanitat i Medi Ambient, Facultat de Farmàcia i Ciències de l’Alimentació, Universitat de Barcelona, Barcelona, Spain; 4 Department of Parasitology, Faculty of Science, Charles University, Prague, Czech Republic

**Keywords:** Anti-sand fly saliva antibodies, Canine leishmaniosis, *Leishmania infantum*, *Phlebotomus perniciosus*, Ibizan hounds, Papular dermatitis, rSP03B

## Abstract

**Background:**

Canine leishmaniosis caused by *Leishmania infantum* is a neglected zoonosis transmitted by sand flies like *Phlebotomus perniciosus*. Clinical signs and disease susceptibility vary according to various factors, including host immune response and breed. In particular, Ibizan hounds appear more resistant. This immunocompetence could be attributed to a more frequent exposure to uninfected sand flies, eliciting a stronger anti-sand fly saliva antibody response.

**Methods:**

This study aimed to investigate the prevalence of anti-*P. perniciosus* saliva antibodies in Ibizan hounds and dogs of other breeds in the *Leishmania*-endemic area of Mallorca, Spain, and to correlate these antibody levels with clinical, immunological and parasitological parameters. Anti-sand fly saliva IgG was examined in 47 Ibizan hounds and 45 dogs of other breeds using three methods: *P. perniciosus* whole salivary gland homogenate (SGH) ELISA; recombinant protein rSP03B ELISA; and rSP03B rapid tests (RT). Additionally, diagnostic performance was evaluated between methods.

**Results:**

Results indicate significantly higher anti-SGH antibodies (*P* = 0.0061) and a trend for more positive SGH ELISA and RT results in Ibizan hounds compared to other breeds. General linear model analysis also found breed to be a significant factor in SGH ELISA units and a marginally significant factor in RT result. Although infection rates were similar between groups, Ibizan hounds included significantly more IFN-γ producers (*P* = 0.0122) and papular dermatitis cases (*P* < 0.0001). Older age and *L. infantum* seropositivity were also considered significant factors in sand fly saliva antibody levels according to at least one test. Fair agreement was found between all three tests, with the highest value between SGH and rSP03B RT.

**Conclusions:**

To our knowledge, this is the first study elaborating the relationship between anti-*P. perniciosus* saliva antibodies and extensive clinical data in dogs in an endemic area. Our results suggest that Ibizan hounds experience a higher frequency of exposure to sand flies and have a stronger cellular immune response to *L. infantum* infection than other breed dogs. Additional sampling is needed to confirm results, but anti-*P. perniciosus* saliva antibodies appear to negatively correlate with susceptibility to *L. infantum* infection and could possibly contribute to the resistance observed in Ibizan hounds.
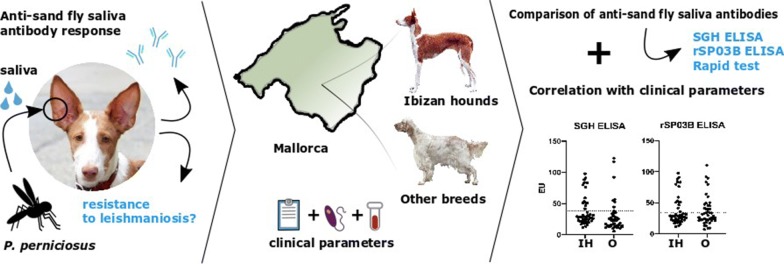

## Background

Canine leishmaniosis (CanL) caused by *Leishmania infantum* is a neglected zoonotic disease endemic in 88 countries around the world, including many parts of the Mediterranean basin [1]. The disease is transmitted by female phlebotomine sand flies, which introduce promastigotes into the host along with various salivary proteins that facilitate infection and induce an immunomodulatory effect [2].

Clinical presentation of CanL is highly variable, manifesting as a subclinical infection, a self-limiting condition, or a debilitating, ultimately fatal disease [3]. In endemic areas, a high proportion of dogs is resistant or subclinically infected with *Leishmania*, with the minority developing serious disease [4]. In particular, a distinctive form of papular dermatitis has been described in infected dogs living in endemic areas, characterized by a lack of other clinical abnormalities, control of parasite dissemination, and favorable disease prognosis [5, 6]. However, all infected dogs, even subclinical dogs, have the potential to serve as a source of infection to sand flies and further transmission [7, 8].

The severity, extent, and initial onset of clinical manifestation is inextricably linked to the host’s immune response. Several studies have demonstrated that protective immunity is associated with a T-cell mediated response, whereas disease susceptibility is associated with a primarily humoral immune response and reduced cell mediated immunity [9, 10]. Although a definite T helper cell 1 (Th1) or T helper cell 2 (Th2) dichotomy was observed in the early studies of leishmaniosis in mice, the immune response to *L. infantum* infection in dogs has proven to be far more complex and more similar to the reactions observed in humans [11]. On a general level, resistant dogs mount a mixed CD4+ T-cell mediated response that favors Th1 activity, producing the pro-inflammatory cytokines interferon gamma (IFN-γ), interleukin 2 (IL-2), and tumor necrosis factor alpha (TNF-α) [12]. These cytokines induce activation of macrophages, which can then kill intracellular amastigotes with nitric oxide (NO), the process of which is considered the main effector mechanism in the protective response [11]. Meanwhile, antibodies are incapable of neutralizing the invading parasites, thus dogs with a strong humoral response are unable to control infection [11].

The type of immune response and associated prognosis can be characterized using a multitude of biomarkers (reviewed in [13]). A common method is the leishmanin skin test (LST), which involves the injection of *Leishmania* antigen to elicit a delayed-type hypersensitivity (DTH) reaction. A positive reaction tends to be associated with non-existent or mild disease and is considered an indicator of favorable prognosis, whereas a low or absent response more commonly occurs in severe cases of CanL [14, 15]. Another method consists of monitoring inflammatory cytokine IFN-γ expression in sera or peripheral blood stimulated with *L. infantum* soluble antigen (LSA). Lack of IFN-γ production has been reported in sick dogs [16], markedly so in dogs with severe disease [17, 18], whereas greater levels of expression have been observed in dogs with subclinical infections or with mild to moderate disease [19, 20]. Diagnosis of papular dermatitis also could be considered an indicator, as the relatively benign condition has been associated with a marked protective cellular immune response and a dampened humoral immune response [5, 6].

Susceptibility to canine leishmaniosis is known to vary depending on a number of host-related factors, including dog breed [21]. Certain dog breeds, such as the boxer, rottweiler, cocker spaniel and German shepherd, have been reported to display a more pronounced susceptibility to the disease due to the influence of numerous loci [22–25]. In contrast, the Ibizan hound is considered more resistant to *L. infantum* infection than dogs of other breeds [26]. It has been proposed that the breed’s relative immunocompetence is derived from a predominantly cellular, parasite-specific immune response, which is associated with a clinically healthy status and favorable prognosis [26, 27]. Compared to other breeds living in the same area, Ibizan hounds display higher frequency of positive LST reactions, greater IFN-γ production, and weaker or lack of humoral immune response [26, 28]. However, a precise etiology for this apparent resistance has not yet been identified.

This study proposes that this distinction in clinical development and comparable immunological competence in the Ibizan hound can be attributed to a greater degree of sand fly exposure. A major defining feature of the breed is its large upright ears, a relatively hairless area especially vulnerable to sand fly bites. Exposure to sand fly bites or immunization with sand fly saliva or its derivatives has been shown to elicit a strong, specific anti-saliva antibody response in dogs [29, 30]. In particular, a positive correlation has been observed between anti-sand fly saliva IgG, IgG1 and IgG2 and the number of sand fly bites for two primary *L. infantum* vectors, the New World *Lutzomyia longipalpis* and the Old World *Phlebotomus perniciosus*; this correlation indicates that this anti-saliva antibody response serves as a marker of sand fly exposure [31, 32]. Moreover, recombinant salivary proteins, such as the yellow-related protein rSP03B of *P. perniciosus*, have been shown to be valid alternatives to traditional salivary gland homogenate (SGH) as a measure of this degree of exposure [33, 34]. Although some studies have shown that anti-*P. perniciosus* saliva antibodies correlate significantly with active canine *L. infantum* infection, as determined by seropositivity for anti-*Leishmania* IgG [32, 33], evidence suggests that exposure to sand fly saliva may be associated with parasite T-cell mediated immunity and favorable clinical outcome [35]. Thus, we hypothesize that the Ibizan hound’s inherently higher frequency of sand fly exposure due to its unique physical attributes could be tied to its later immunocompetence during *L. infantum* infection.

Therefore, the objectives of this study were to investigate the prevalence of anti-*P. perniciosus* saliva antibodies in Ibizan hounds and dogs of other breeds living in a highly endemic area of leishmaniosis and to correlate these anti-saliva antibody levels with clinical, immunological and parasitological parameters. In particular, the relationship between papular dermatitis suggestive of *L. infantum* infection and anti-saliva antibodies was examined. To our knowledge, this is the first study attempting to elaborate the relationship between anti-*P. perniciosus* saliva antibodies and extensive clinical data in dogs residing in an endemic area, aside from previous studies considering primarily serological status for *L. infantum*.

## Methods

### Experimental design and clinical data

A cross-sectional study was conducted in December during three consecutive years (2014–2016) on the island of Mallorca, Spain. From five different herds of hunting dogs, 47 samples were collected from Ibizan hounds and 45 from dogs of other breeds, all of which were living in outdoor kennels (including sleeping outdoors) in inland villages under similar environmental conditions (*n* = 92 dogs). The majority of samples (71 dogs) were obtained in 2015, whereas a small portion of the Ibizan hound samples were taken in 2014 and 2016 (13 and 7 dogs, respectively). Basic clinical characteristics were recorded, including breed, sex and age. Dogs did not receive any form of treatment for leishmaniosis in the years preceding the study, with the exception of a single non-Ibizan hound dog, who had been treated with allopurinol 2.5 years prior to sample collection. Ibizan hounds did not receive any insecticide treatment, but a few dogs of other breeds were reported to have received some topical individual insecticide treatment (permethrin spot-on or collar), albeit with inconsistent application and low owner compliance with recommended guidelines. Dogs did not have any history of travel outside Mallorca. All dogs were subjected to a complete physical examination, with all clinical signs recorded. In addition, specific dermatological examination was performed. Raised, solid cutaneous lesions in non-haired skin with a width less than one centimeter (papules) or with a greater width than height (plaques) with or without an ulcerative or crusted central part (volcano sign) were considered suggestive of papular dermatitis due to leishmaniosis [5, 6]. In addition, a sample was collected for cytological and molecular examination if the size of the lesion was sufficient, with qPCR for *L. infantum* applied if presence of *Leishmania* amastigotes was unclear [36].

Additional diagnostic procedures previously performed and described elsewhere consisted of in-house endpoint indirect enzyme-linked immunosorbent assay (ELISA) for detection of anti-*L. infantum* antibodies (IgG) [37], LST [26], *L. infantum* quantitative PCR (qPCR) of DNA extracted from peripheral blood [37], and IFN-γ assay using whole peripheral blood stimulated with LSA [17]. Not all diagnostic tests were performed for all dogs due to lack of sample and/or lack of clinical justification. Residual blood and serum samples were used in this study, thus ethical approval was not needed.

Fifty-seven control residual serum samples were obtained from the Royal Veterinary College (London, UK) from a random set of dogs presenting at the Queen Mother Veterinary Teaching Hospital. These dogs were assumed to be unexposed to sand flies because they were obtained from a non-endemic region for CanL. No clinical information or travel history was available for the UK dogs.

### Indirect enzyme-linked immunosorbent assay (ELISA)

Sera samples were analyzed by indirect ELISA measuring anti-*P. perniciosus* IgG by using *P. perniciosus* salivary gland homogenate (SGH) and the recombinant protein rSP03B as the antigen. ELISAs were performed in accordance with a previous study [38]. Absorbance (OD) was measured at 492 nm by using an automatic reader (Heales MB-580, Shenzhen, China). All sera samples were tested in duplicate. Due to limited rSP03B availability, only the first 40 of the 57 UK control samples were used for the rSP03B ELISA and RT, whereas all 57 UK samples were subjected to the SGH ELISA.

As plates were analyzed on different days, positive control sera (PC, *n* = 2) and negative control sera (NC, *n* = 1) were included on each plate. A conjugate control (CC), or blank, containing no sera was included in each plate. To control for inter-plate variability, ELISA units (EU) were calculated as the standardized optical density (SOD) as follows:

$$SOD\,\left( {\text{\%}} \right) = \frac{{Average \, OD_{Sample} - Average \, OD_{CC} }}{{Average \, OD_{PC} - Average \, OD_{CC} }} \times 100$$

Cut-off SOD values were established with the package OptimalCutpoints [39] for Receiver Operating Characteristic (ROC) curve analysis in R software [40]. The ROC curves were generated and compared by two common methods, the Youden index [41] and “SpEqualSe”, the latter of which minimizes the absolute value of the difference between sensitivity and specificity [42–44]. The “true” disease status of samples included in the calculation of the ROC curve was determined by using the average SOD of the negative control UK sera samples for the SHG ELISA, as this test was previously demonstrated to have greater sensitivity and specificity than either test employing the recombinant rSP03B [38]. Samples with an OD above three standard deviations of the mean SGH ELISA SOD for the UK samples were considered highly likely to have been exposed to sand flies and included to approximate “true positive” samples in the calibration. All UK samples were classified as “true negative” samples, except for two samples exceeding three standard deviations (SD) of the mean. Due to lack of clinical information and travel history, these two dogs were excluded from the calibration. The remaining sera samples from Mallorcan dogs that were within three SD of the mean UK SOD were considered “indeterminate status” and thus excluded from the calculation for calibrating the ROC curve. Finally, a single recommended cut-off value of 38.05 EU was established by both methods for SGH. The analyses suggested three different cut-off values with identical area under the curve (AUC) for rSP03B ELISA; from these values, the middle value balancing sensitivity and specificity was chosen (33.67 EU).

### Rapid test with rSP03B sero-strip

Sero-strip tests were completed as described elsewhere [38]. Tests were conducted with all serum samples from Mallorca, as well as with the 34 of 57 UK samples that were included in both the SGH and rSP03B ELISA analysis. Results were visually examined and photographed after precisely 15 min of allowing the migration buffer to run. For comparison, a positive control serum was tested on each day that rapid tests were conducted.

Results from the rSP03B sero-strip test were categorized according to the intensity of the observed band and assigned one of five values: 0 for a lack of band and negative result; 1, for a very faint band of indeterminate nature; 2, for a weak but positive band; 3, for a very clear positive band of a weaker intensity than the control band; and 4, for a strong positive band comparable in intensity to the control band [38]. Only samples receiving a score above 1, rather than at least 1, were considered positive, a modification from the original protocol suggested to improve performance of the RT in the field [38]. An example spectrum of results is provided as part of the additional files (Additional file 1: Figure S1).

### Statistical analyses

Statistical tests were performed with a combination of Minitab 17 and R software [40, 45]. Diagnostic performance was evaluated by comparing degree of agreement between the three salivary antibody tests performed. McNemar’s test and Cohen’s kappa were calculated for each pair of diagnostic tests, the latter of which was assessed according to the original author’s specifications [46]. Clinical parameters were analyzed between the two sample groups (Ibizan hounds and dogs of other breeds) using a Mann–Whitney U-test or Fisher’s exact test as appropriate. Due to the small size of the data set, a normal approximation for the Fisher’s exact tests was not used in most cases. Any differences in EU for SGH or rSP03B between groups based on clinical parameters were determined using two-sided Mann–Whitney U-tests. A one-sided Fisher’s exact test was then used to examine the significance of the trend established when calculating the proportion of positive ELISA or RT results for each variable. Spearman’s correlation was used to determine any association between SGH and rSP03B EU and quantitative clinical variables. A *P*-value of less than 0.05 was considered significant.

A multivariate general linear model (GLM) was constructed for each salivary antigen test using R software, including only the quantitative and qualitative variables with a *P*-value of less than 0.2 based on univariate analysis. Five models were created in total, one each to predict the following response variables: SGH ELISA result; SGH EU; rSP03B ELISA result; rSP03B EU; and rSP03B RT result. All models predicting test result were constructed using binomial logistic models, to predict the probability being classified as positive for that particular test (i.e. SGH ELISA result, rSP03B ELISA result and RT result). SGH and rSP03B EU were predicted using Gaussian linear regression models. All EU (i.e. for the SGH, rSP03B, IFN-γ, and *L. infantum* ELISA) were calculated as the SOD using the formula previously provided and log_10_-transformed before GLM analysis. To examine our primary hypothesis, breed category (Ibizan hound or other breed) was included as a variable in the final multivariate models irrespective of its significance, or lack thereof, in the previous univariate models. When considering variables with a high degree of multicollinearity, such as *L. infantum* seropositivity status and *L. infantum* EU, the variable with the lowest *P*-value in the univariate analysis was retained in the multivariate model. Multivariate models were constructed in a stepwise fashion, beginning with a full model and removing variables one-by-one. Multiple models for the same response variable were compared using the Akaike’s information criterion (AIC). Goodness-of-fit was assessed by deviance of the residuals, ruling out overdispersion. After simplifying the models, interactions between selected variables were considered. Non-significant interactions were included so long as they improved model fit. Final models were compared using a likelihood ratio test.

## Results

### Description of clinical data for Ibizan hounds and dogs of other breeds

#### Dogs

Samples were collected from 47 Ibizan hounds and 45 dogs of other breeds (*n* = 92), all sexually intact. The non-Ibizan hound group included 30 purebred dogs, of which the most frequent breeds were English setter (*n = *12), Brittany spaniel (*n* = 10), Weimaraner (*n* = 2), and shorthaired pointer (*n* = 2). Fifteen mixed breed dogs were also represented, derived from crosses between other hunting or working breeds. The median age of the entire sample set was 24 months (range 4–132 months; 7 dogs without age recorded), with 62.35% of the dogs above 18 months of age. A significant difference in age was detected between the two groups: the Ibizan hound group was characterized by a significantly lower median age and lower proportion of adult dogs (median: 12 months; 18/40 or 45% adults) in comparison with the group of other breeds (median: 42 months; 35/45 or 77.78% adults; Mann–Whitney U-test: *U* = 1432, *P = *0.0113; Fisher’s exact test: *P = *0.0033). The proportion of dogs at least 4 years-old also was statistically different between Ibizan hounds (9/40 or 22.5%) and other breeds (20/45 or 44.44%; Fisher’s exact test: *P = *0.0408). Both Ibizan hounds (12/47 or 74.47%) and other breeds (20/45 or 55.56%) consisted of a greater proportion of females, but the difference in the proportion of females between the two groups was not statistically significant (Fisher’s exact test: *P = *0.0797).

#### *Leishmania infantum* infection status

Table 1 summarizes the proportion of positive results for the four primary diagnostic tests for *L. infantum* infection between breed groups. No difference was detected in *L. infantum* antibody levels (EU) or in the proportion testing positive when comparing Ibizan hounds and other breeds. Moreover, the overwhelming majority of positive results were considered “low positive” (EU > 100 and < 500), except for three “medium positives” (EU > 500 and < 9000) observed in non-Ibizan hound dogs. Based on *L. infantum* qPCR from blood, the proportion of Ibizan hounds classified as positive (35.29%) was similar to that observed in dogs of other breeds (51.22%). However, blood parasite load was significantly greater in dogs of other breeds (median 0.33 parasites/ml) than in Ibizan hounds (median 0 parasites/ml; Mann–Whitney U-test: *U* = 1099, *P* = 0.0240). Although no significant difference was detected in the proportion of LST-positive dogs, Ibizan hounds displayed significantly greater LST lesion diameter and skin thickness (medians 2.1 mm and 5.48 mm, respectively) in comparison with dogs of other breeds (medians 1.0 mm and 3.45 mm, respectively; Mann–Whitney U-test: *U* = 885.5 *P* = 0.0002 and *U* = 946.5, *P* = 0.0001, respectively). A significant difference between the two groups was observed in the proportion of dogs classified as IFN-γ producers (Fisher’s exact test: *P* = 0.0122), with a greater proportion observed in Ibizan hounds (35/39 or 89.74%) than other breeds (16/26 or 61.54%). Likewise, IFN-γ levels were significantly higher in Ibizan hounds (median 1115.60 EU) than in other breeds (median: 51 EU; Mann–Whitney U-test: *U* = 1506, *P* = 0.0017). No significant difference in infection rate was found between groups, as determined by the proportion of dogs testing positive for any of the four tests listed in Table 1.

**Table 1:** Proportion of dogs testing positive for the four main diagnostic tests for confirming *L. infantum* infection

Positive diagnostic test	Proportion of Ibizan hounds (*n* = 47)	Proportion of other breeds (*n* = 45)	*P*-value
*L. infantum* ELISA	20% (9/45)	22.22% (10/45)	1
*L. infantum* blood qPCR	35.29% (12/34)	51.22% (21/41)	0.2427
LST	93.55% (29/31)	76.19% (16/21)	0.1038
IFN-γ assay	89.74% (35/39)	61.54% (16/26)	0.0122*
Any of four tests	91.11% (41/45)	75.56% (34/45)	0.0874

*Note*: Two-tailed Fisher’s exact test

*P < 0.05

*Abbreviations*: ELISA, enzyme-linked immunosorbent assay; IFN-γ, interferon gamma; LST, leishmanin skin test; qPCR, quantitative polymerase chain reaction

#### Diagnosis of papular dermatitis

Single or multiple papules were located in the inner aspect of one or both pinna (Additional file 2: Figure S2). In the majority of dogs, these papules were centrally ulcerated and covered by an adherent crust. No other cutaneous lesions were observed, aside from mild crusting dermatitis suggestive of fly dermatitis in the pinnae of two Ibizan hounds. Significantly more Ibizan hounds (30/47 or 63.83%) than dogs of other breeds (1/45 or 2.22%) presented with papular dermatitis suggestive of *L. infantum* infection (Fisher’s exact test, *P *< 0.0001). A single non-Ibizan hound presented with clinical signs consistent with diagnosis of papular dermatitis, but later cytological examination of the papule suggested that it could be due to an eosinophilic arthropod bite reaction, as no amastigotes were detected and it did not display the typical “volcanic” appearance (Additional file 2: Figure S2). This dog was confirmed as infected by low positive *L. infantum* serology and a positive reaction to LST, although it received a negative result for the IFN-γ assay and the *L. infantum* qPCR in blood. Subsequent statistical analyses examining the anti-saliva antibody response between papular and non-papular cases included this dog among the dogs with papular dermatitis, except where noted.

Of the 17 Ibizan hounds without papules, 15 were considered positive for *L. infantum* infection. Etiology of papular lesions was confirmed in 18 Ibizan hounds (definitive diagnosis) by means of visualization of amastigotes on skin samples (*n* = 9) or by molecular detection in stained smears (*n* = 9). As determined by these methods, the proportion of dogs with a definitive diagnosis of papular dermatitis due to *Leishmania* infection was statistically significant between breed groups (Fisher’s exact test: *P *< 0.0001).

There was slight agreement between papular dermatitis diagnosis and IFN-γ assay or LST (κ = 0.099 and 0.055, respectively). However, when considering only dogs less than 18 months-old, the degree of agreement between papular dermatitis diagnosis and IFN-γ assay or LST was moderate (κ** = **0.479 and 0.42, respectively).

### Evaluation of the diagnostic performance and agreement between SHG ELISA, rSP03B ELISA, and rSP03B rapid sero-strip test

**Fig. 1:**
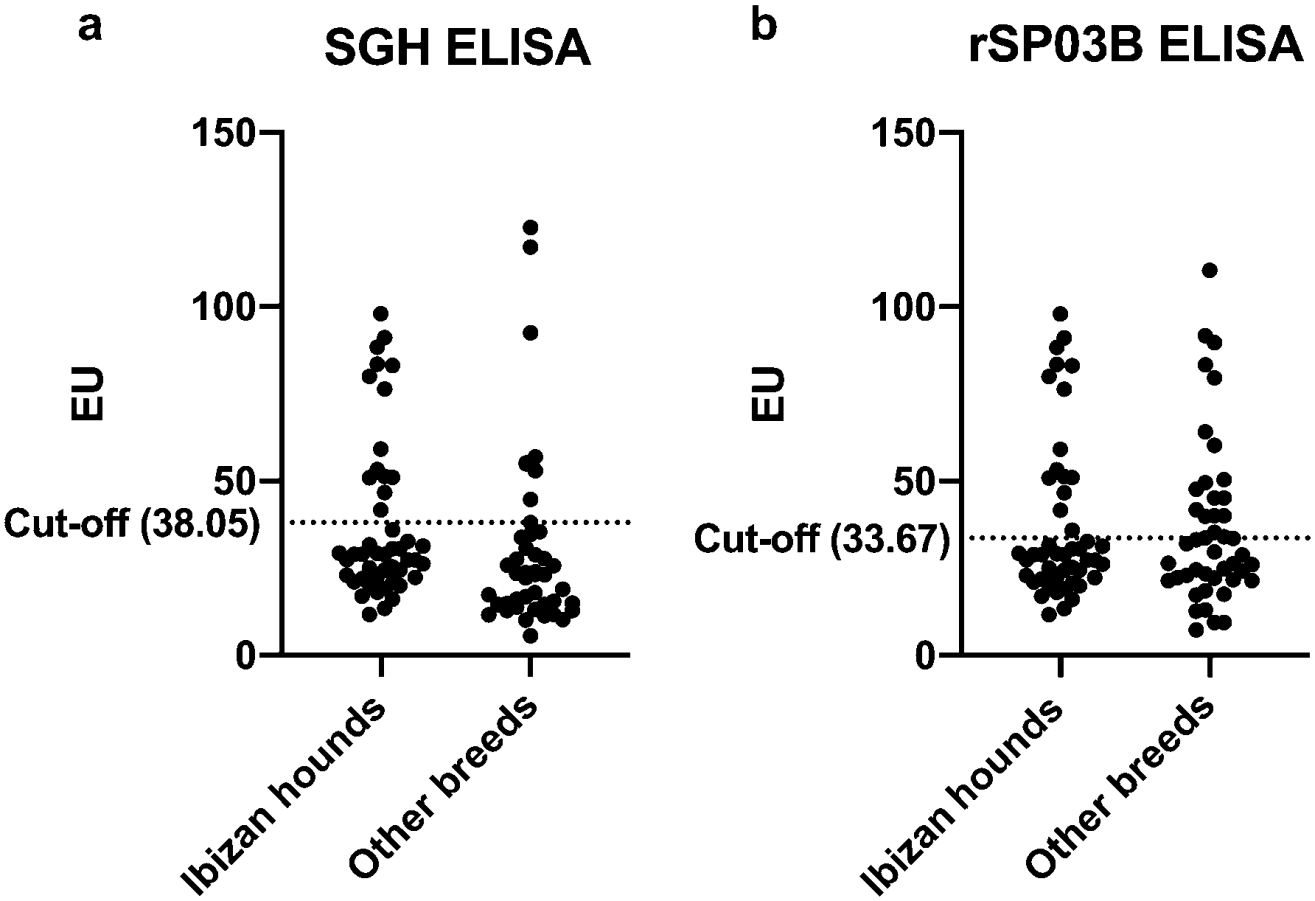
Distribution of SGH ELISA (**a**) and rSP03B ELISA (**b**) EU based on breed. Respective cut-offs are indicated by a dotted line

**Fig. 2:**
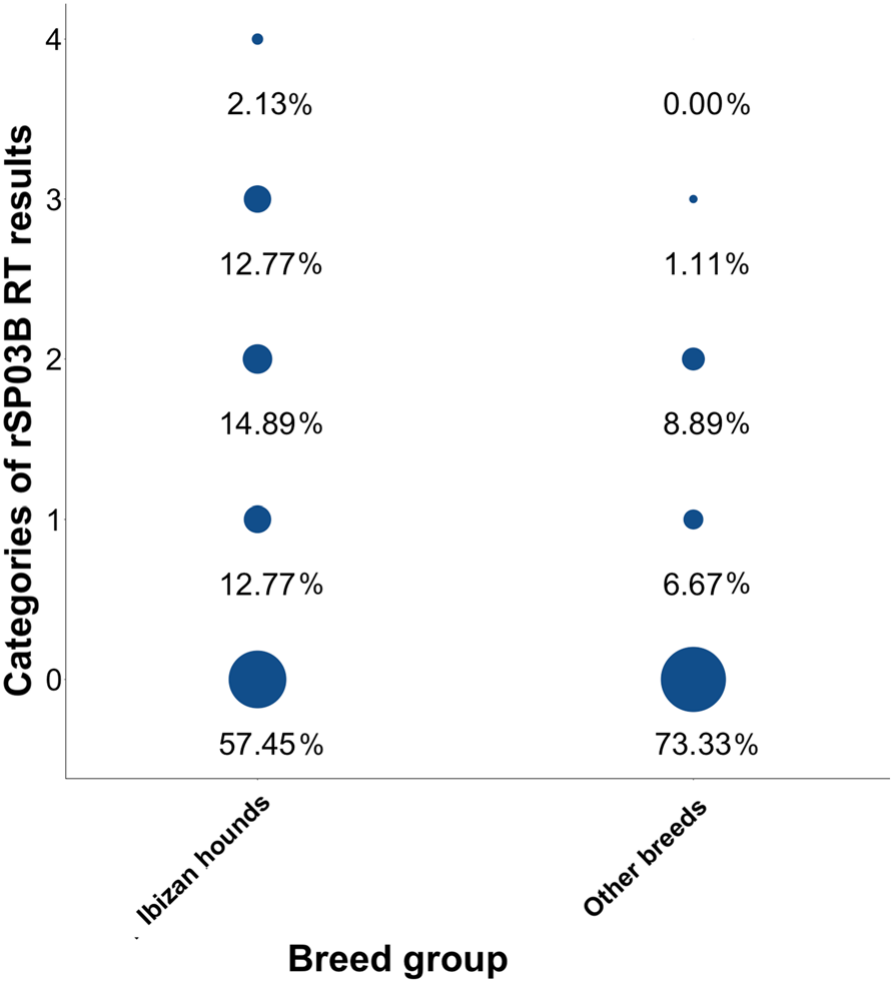
Distribution of RT score categories based on breed. Only scores above one are considered positive. The percentage of dogs corresponding to each category is listed below the appropriate dot

Pairwise analysis was used to examine agreement between the three salivary antigen-based tests, the results of which are depicted in Table 2. Figures 1 and 2 depict the distribution of SGH and rSP03B ELISA EU and of RT categories, respectively, based on breed group. Correlation between SGH and rSP03B EU is shown in Table 3. Additional data on test score differences between breed groups are provided in Tables 4, 5 and 6 (SGH ELISA, rSP03B ELISA and RT, respectively). A relatively high percentage of agreement (range 73.48–81.06%) and fair degree of agreement (κ range: 0.3312–0.3846) was observed between each of the three pairs of tests. Highest percentage of agreement was observed between SGH ELISA and RT (81.06%, κ** = **0.3736 ± 0.1014), while highest degree of agreement was reported between SGH and rSP03B ELISA (75.76%, κ** = **0.3846 ± 0.0840**)**. However, a statistically significant difference was found in the proportions of positive responses between the SGH ELISA (23/92 or 25%) and rSP03B ELISA (33/92 or 35.87%; McNemar’s test: *P = *0.0007) and between rSP03B ELISA and RT (23/92 or 25%; McNemar’s test: *P = *0.0012). Interestingly, a moderate but significant Spearman’s correlation of 0.4759 (*df* = 90, *P *< 0.0001) was obtained when evaluating the EU for the two ELISA tests, as depicted in Table 3.

**Table 2:** Comparison of agreement between salivary antibody tests

Test pair	Percent agreement	McNemar’s exact test *P*-value	κ ± SE	κ interpretation^a^
SGH ELISA *vs* rSP03B ELISA	75.76%	0.0007***	0.3846 ± 0.0840	Fair
SGH ELISA *vs* rSP03B RT	81.06%	0.8450	0.3736 ± 0.1014	Fair
rSP03B ELISA *vs* rSP03B RT	73.48%	0.0012**	0.3312 ± 0.0855	Fair

^a^The interpretation for each κ value is shown in the final column according to the following scale: ≤ 0, no agreement; 0.01–0.20, none to slight; 0.21–0.40, fair; 0.41– 0.60, moderate; 0.61–0.80, substantial; and 0.81–1.00, almost perfect agreement

**P < 0.01, ***P < 0.001

*Abbreviations*: ELISA, enzyme-linked immunosorbent assay; κ, Cohen’s kappa value; RT, rapid test; SE, standard error; SGH, salivary gland homogenate

**Table 3:** Determination of the relationship between salivary antigen EU and other quantitative clinical parameters using Spearman’s correlation

Relationship	SGH ELISA		rSP03B ELISA	
	*r*_*s*_	*P*-value	*r*_*s*_	*P*-value
Salivary antigen EU and LST diameter (mm)	0.0625	0.6729	− 0.1177	0.4258
Salivary antigen EU and LST thickness (mm)	0.0958	0.5081	− 0.1669	0.2468
Salivary antigen EU and *L. infantum* EU	0.3585	0.0005***	0.3888	0.0002***
Salivary antigen EU and IFN-γ antigen EU	0.2331	0.0616	0.0644	0.6104
Salivary antigen EU and blood parasite load (parasites/ml)	0.0974	0.4056	0.1876	0.1070
Salivary antigen EU and age (months)	0.2360	0.0296*	0.5212	< 0.0001***
SGH and rSP03B EU	0.4759	< 0.0001***		

*Abbreviations*: ELISA, enzyme-linked immunosorbent assay; EU, ELISA units; IFN-γ, interferon gamma; LST, leishmanin skin test; *r*_*s*_, Spearman’s rho coefficient; SGH, salivary gland homogenate

*P < 0.05, ***P < 0.001

**Table 4:** Comparison of SGH EU and proportion of positive results between dogs based on clinical parameters

Variable	*n*	SGH EU		SGH ELISA result	
		Median ELISA units (IQR)	Mann–Whitney *P*-value	Proportion positive (count)	Fisher’s exact test *P*-value
Breed					
Ibizan hounds	47	28.94 (19.76)	0.0061**	0.2979 (14)	0.1999
Other breeds	45	23.07 (19.76)		0.200 (9)	
Age					
Adult (≥ 18 months)	53	26.13 (24.81)	0.255	0.2830 (16)	0.1426
Juvenile (< 18months)	32	23.32 (17.06)		0.1563 (5)	
Sex					
Female	60	25.48 (16.52)	0.5633	0.2333 (14)	0.3957
Male	32	26.54 (35.44)		0.2813 (9)	
*L. infantum* ELISA					
Seropositive	19	28.94 (35.05)	0.1953	0.4211 (8)	0.0470*
Seronegative	71	25.25 (16.56)		0.1972 (14)	
*L. infantum* blood qPCR					
Positive	33	27.62 (35.41)	0.3503	0.3030 (10)	0.1310
Negative	42	24.18 (13.93)		0.1667 (7)	
LST					
Positive	45	24.40 (13.39)	0.1715	0.1778 (8)	0.2864
Negative	7	23.69 (13.24)		0 (0)	
IFN-γ assay					
Producer	51	25.25 (20.76)	0.1088	0.2590 (13)	0.3116
Non-producer	14	19.34 (16.36)		0.1429 (2)	
Papule presence					
Papular	31	26.13 (26.35)	0.2980	0.2581 (8)	0.5450
Non-papular	61	25.70 (21.56)		0.2459 (15)	
*L. infantum* infection^a^					
Yes	75	27.29 (26.70)	0.0832	0.2800 (21)	0.0689
No	15	23.69 (14.93)		0.0667 (1)	

^a^Presence of *L. infantum* infection defined as positive result for at least one of the following tests: *L. infantum* ELISA, *L. infantum* blood qPCR, IFN-ɣ assay, or LST. Only dogs subjected to at least one of the tests are considered

*Notes*: Mann–Whitney tests were all conducted as two-tailed analyses, while Fisher’s exact tests were all single-tailed, examining the trend established when calculating the proportion of positive results

*Abbreviations*: ELISA, enzyme-linked immunosorbent assay; EU, ELISA units; IFN-γ, interferon gamma; IQR, interquartile range; LST, leishmanin skin test; qPCR, quantitative polymerase chain reaction; SGH, salivary gland homogenate; n, number of dogs

*P < 0.05, **P < 0.01

**Table 5:** Comparison of recombinant protein rSP03B EU and proportion of positive results between dogs based on clinical parameters

Variable	*n*	rSP03B EU		rSP03B ELISA result	
		Median ELISA units (IQR)	Mann–Whitney *P*-value	Proportion positive (count)	Fisher’s exact test *P*-value
Breed					
Ibizan hounds	47	25.63 (22.64)	0.1668	0.2979 (14)	0.1525
Other breeds	45	29.74 (23.27)		0.4222 (19)	
Age					
Adult (≥ 18 months)	53	33.17 (24.41)	0.0001***	0.4528 (24)	0.0021**
Juvenile (< 18months)	32	20.93 (15.56)		0.1250 (4)	
Sex					
Female	60	25.64 (22.26)	0.1672	0.3333 (20)	0.3188
Male	32	30.44 (24.20)		0.4063 (13)	
*L. infantum* ELISA					
Seropositive	19	34.10 (38.74)	0.0275*	0.5263 (10)	0.0709
Seronegative	71	25.63 (21.04)		0.3099 (22)	
*L. infantum* blood qPCR					
Positive	33	29.74 (19.26)	0.2787	0.3939 (13)	0.3016
Negative	42	24.32 (22.44)		0.3095 (13)	
LST					
Positive	45	23.69 (14.64)	0.5199	0.2222 (10)	0.5164
Negative	7	24.96 (23.73)		0.2857 (2)	
IFN-γ assay					
Producer	51	25.63 (16.83)	0.7196	0.2549 (13)	0.1742
Non-producer	14	23.19 (48.24)		0.4229 (6)	
Papule presence					
Papular	31	25.18 (20.78)	0.1554	0.3226 (10)	0.3906
Non-papular	61	26.87 (23.68)		0.3770 (23)	
*L. infantum* infection^a^					
Yes	75	26.38 (19.39)	0.8795	0.3467 (26)	0.4533
No	15	24.96 (29.19)		0.4000 (6)	

^a^Presence of *L. infantum* infection defined as positive result for at least one of the following tests: *L. infantum* ELISA, *L. infantum* blood qPCR, IFN-γ assay, or LST. Only dogs subjected to at least one of the tests are considered

*Notes*: Mann–Whitney tests were all conducted as two-tailed analyses, while Fisher exact tests were all single-tailed, examining the trend established when calculating the proportion of positive results

*Abbreviations*: ELISA, enzyme-linked immunosorbent assay; EU, ELISA units; IFN-γ, interferon gamma; IQR, interquartile range; LST, leishmanin skin test; qPCR, quantitative polymerase chain reaction; n, number of dogs

*P < 0.05, **P < 0.01, ***P < 0.001

**Table 6:** Comparison of the proportion of positive rSP03B RT results between dogs based on clinical parameters

Variable	*n*	rSP03B RT result	
		Proportion positive (count)	Fisher’s exact test *P*-value
Breed			
Ibizan hounds	47	0.2978 (14)	0.1999
Other breeds	45	0.2000 (9)	
Age			
Adult (≥ 18 months)	53	0.3208 (17)	0.0140*
Juvenile (< 18 months)	32	0.0938 (3)	
Sex			
Female	60	0.2667 (16)	0.4053
Male	32	0.2188 (7)	
*L. infantum* ELISA			
Seropositive	19	0.3684 (7)	0.1333
Seronegative	71	0.2113 (15)	
*L. infantum* blood qPCR			
Positive	33	0.3030 (10)	0.1945
Negative	42	0.1905 (8)	
LST			
Positive	45	0.2000 (9)	0.1917
Negative	7	0.4286 (3)	
IFN-γ assay			
Producer	51	0.2353 (12)	0.3674
Non-producer	14	0.3571 (5)	
Papule presence			
Papular	31	0.2258 (7)	0.4550
Non-papular	61	0.2623 (16)	
*L. infantum* infection^a^			
	75	0.2400 (18)	0.5286
	15	0.2667 (4)	

^a^Presence of *L. infantum* infection defined as positive result for at least one of the following tests: *L. infantum* ELISA, *L. infantum* blood qPCR, IFN-γ assay, or LST. Only dogs subjected to at least one of the tests are considered

*Notes*: Fisher’s exact tests were conducted as single-tailed analyses, examining the trend established when calculating the proportion of positive result

*Abbreviations*: ELISA, enzyme-linked immunosorbent assay; IFN-γ, interferon gamma; IQR, interquartile range; LST, leishmanin skin test; qPCR, quantitative polymerase chain reaction; RT, rapid test; n, number of dogs

*P < 0.05

### Relationship between *P. perniciosus* salivary antibodies and clinical parameters

Table 3 describes the correlation between individual clinical quantitative variables and SGH and rSP03B EU. SGH ELISA, rSP03B ELISA and rSP03B RT outcomes (EU and/or results) are summarized by individual clinical parameters in Tables 4, 5 and 6, respectively.

#### Breed

Median SGH EU appeared higher for Ibizan hounds (28.94) than for dogs of other breeds (23.07), and further analysis found the difference between breed groups to be significant (Mann–Whitney U-test: *U* = 2537, *P = *0.0061).

#### Age

A significant positive correlation was reported between age and SGH EU or rSP03B EU (Spearman’s correlation, SGH: *r*_*s* = _0.2360, *df* = 83, *P* = 0.0296; rSP03B: *r*_*s *_= 0.5212, *df*  = 83 *P < *0.0001). Adult dog rSP03B EU was considered to be significantly different from juvenile dog EU (Mann–Whitney U-test: *U* = 2700, *P = *0.0001). This trend correlated to a significantly higher proportion of positive results in adults (26/53 or 45.23%) than in juvenile dogs (4/32 or 12.5%; Fisher’s exact test: *P = *0.0019). Likewise, a significantly higher proportion of positive RT results was observed in adult dogs (17/53 or 32.08%) than in juvenile dogs (3/32 or 9.38%; Fisher’s exact test: *P = *0.0140).

#### *Leishmania infantum* serological status

Dogs seropositive for *L. infantum* were significantly more likely to be classified as seropositive for anti-SGH antibodies (Fisher’s exact test: *P = *0.0470). A statistically significant difference in SP03B EU was found between *L. infantum*-positive dogs (median 34.10) and negative dogs (median 25.63; Mann–Whitney U-test: *U* = 3007, *P = *0.0275). Additionally, a moderately strong, positive correlation was described between *L. infantum* EU and SGH or rSP03B EU (Spearman’s correlation, SGH: *r*_*s*_ =  0.3585, *df* = 86, *P = *0.0005; rSP03B: *r*_*s*_ =  0.3888, *df* = 86, *P = *0.0002).

### Multivariate general linear models (GLMs)

Final GLM results are summarized in Table 7. After removing variables with a high degree of multicollinearity and simplifying the model to maximize the fit, only two additional variables were considered significant in any of the models: *L. infantum* EU and age in months. Additionally, an interaction between breed group and age (Age × Breed) was considered to account for the statistically significant difference in age in Ibizan hounds and dogs of other breeds. Although this interaction was not considered significant in any model, it was retained in four of five models because it improved fit. RT was the only test for which model fit was not improved by including this interaction. After adjustment for the age difference between breed groups (Age × Breed), breed was considered a significant factor in predicting SGH EU (*P = *0.0026), with Ibizan hounds more likely to have higher EU. Breed did not quite reach significance in predicting RT result (*P = *0.0745). *Leishmania infantum* EU was considered a significant variable in all of the models created except for RT. Age (months) was also considered significantly related with EU and/or result in all models.

**Table 7:** Estimates of the multivariate models of the relationship between the outcome of the three salivary antigen tests (EU and/or result) and clinical parameters

Model	Factor	Estimate	SE	*P*-value	Missing observations
SGH result	Breed (Ibizan hound)	1.8057	1.6223	0.1424	0
	*L. infantum* EU^a^	1.8612	0.7809	0.0171*	2
	Age (months)	0.0282	0.0139	0.0423*	7
	Age × Breed^b^	− 0.0208	0.0201	0.3024	
SGH EU^a^	Breed (Ibizan hound)	0.2562	0.0824	0.0026**	0
	*L. infantum* EU^a^	0.1738	0.0709	0.0164*	2
	Age (months)	0.0032	0.0010	0.0028**	7
	Age × Breed^b^	− 0.0029	0.0017	0.1057	
rSP03B ELISA result	Breed (Ibizan hound)	− 0.2759	0.9843	0.7793	0
	*L. infantum* EU^a^	1.4593	0.7317	0.0461*	2
	Age (months)	0.0295	0.0113	0.0093**	7
	Age × Breed^b^	− 0.0118	0.0184	0.5221	
rSP03B EU^a^	Breed (Ibizan hound)	− 0.0018	0.0704	0.9792	0
	*L. infantum* EU^a^	0.1812	0.0605	0.0037**	2
	Age (months)	0.0037	0.0009	0.0001***	7
	Age × Breed^b^	− 0.0013	0.0015	0.8660	
RT result	Breed (Ibizan hound)	1.0991	0.6163	0.0745	0
	Age (months)	0.0259	0.0086	0.0003**	7

^a^All EU (SGH, rSP03B, and *L. infantum*) were log_10_ transformed prior to modeling

^b^Interaction between age (months) and breed group

*Abbreviations*: ELISA, enzyme-linked immunosorbent assay; EU, ELISA units; GLM, general linear model; IFN-ɣ, interferon gamma; IQR, interquartile range; LST, leishmanin skin test; qPCR, quantitative polymerase chain reaction; RT, rapid test; SE, standard error; SGH, salivary gland homogenate

*P < 0.05, **P < 0.01, ***P < 0.001

## Discussion

Canine leishmaniosis caused by *L. infantum* is a complex zoonosis that frequently eludes definitive diagnosis and manifests in a spectrum of disease depending on a variety of host characteristics that are not yet entirely understood [47]. Although breed and host immune response are both established as key factors in an individual’s susceptibility to disease, it is unclear how these two are related [11, 13]. In order to address this knowledge gap, this study examined anti-*P. perniciosus* saliva antibodies in typically *L. infantum-*resistant Ibizan hounds [26] and in dogs of other breeds. Whereas previous epidemiological field studies have considered primarily seropositivity for *L. infantum* or a single measure of the anti-*P. perniciosus* saliva response [29, 33], this study examined multiple additional clinical parameters in relation to anti-*P. perniciosus* antibodies, quantified by means of three different methods.

Our results suggest that the anti-saliva antibody response differed between Ibizan hounds and dogs of other breeds. As determined by SGH ELISA, a significant difference was detected in anti-*P. perniciosus* antibodies in Ibizan hounds in comparison with other breeds of dogs living in the same geographical area under similar environmental conditions. Although it failed to reach significance, a trend for a greater proportion of positive SGH ELISA and rSP03B RT results also was observed for Ibizan hounds. Because the level of anti-sand fly saliva antibodies is reflective of the intensity of vector exposure [29, 32, 34, 48, 49], these findings might imply a higher frequency of sand fly bites in Ibizan hounds. It is unlikely that this difference in sand fly biting frequency is due to different environmental conditions between groups; all dogs slept outdoors (outdoor kennels) and received either no or very sparse, inconsistent insecticide treatment, both factors which individually predispose dogs to a high degree of vector exposure [50]. In contrast with the more pendular ears of the other breed group, the erected pinnae of Ibizan hounds offer an easily accessible contact area devoid of hair, a characteristic that could predispose the breed to phlebotomine bites.

Studies in rodent and dog models have demonstrated that prior, repeated exposure to sand fly bites or immunization with sand fly saliva or its derivatives is associated with reduced disease severity in cutaneous or visceral leishmaniosis [2, 35, 51]. This protective effect was later shown by several studies to derive from the induction of a strong Th1-dominant cellular immune response, characterized by DTH reaction and increased production of IFN-γ and/or IL-12 [2]. It could be hypothesized that the repeated, high exposure to sand fly bites experienced by Ibizan hounds could induce a similar immune response upon infection with *L. infantum*, possibly contributing to their comparative immunocompetence.

Our findings appear to support that Ibizan hounds are more resistant to CanL and that this resistance is likely tied to a strong, parasite-specific cellular immune response. Ibizan hounds displayed a significantly higher rate of papular dermatitis clinically suggestive of *L. infantum* infection when compared with dogs of other breeds in a highly endemic area of CanL. In particular, 63.83% (30/47) of Ibizan hounds presented with single or multiple papules, whereas only 2.22% (1/45) of the other dogs had a single papulo-crusting lesion. This difference remained significant even when considering only papular dermatitis cases with confirmed *L. infantum* infection. Although this increased prevalence might be attributed to a higher rate of *L. infantum* infection, this study found similar rates of infection between the two groups (91.11% of Ibizan hounds and 75.56% of other breed dogs), as defined by a positive result for at least one of the four associated tests (i.e. *L. infantum* ELISA, *L. infantum* qPCR of blood, IFN-γ assay, or LST). The host immune response is known to influence clinical presentation of CanL [11], thus the higher prevalence of papular dermatitis observed in the Ibizan hounds could be related to a divergence in the immune response mounted against *L. infantum* infection. Indeed, in the present study, Ibizan hounds contained significantly more IFN-γ producers and produced significantly higher levels of IFN-γ than other breeds. This result aligns well with a previous work, which reported marked *Leishmania*-specific IFN-γ production in healthy Ibizan hounds when compared to control and sick dogs [28]. In general, production of this pro-inflammatory cytokine is correlated with disease resistance or mild disease [12, 19, 20, 52], while lack of expression is described in experimentally infected symptomatic dogs [12] and in dogs with a strong humoral response and severe disease [17, 18]. Contrary to expectations, the proportion of positive LST reactions was not substantially different between groups, although a previous study described a higher frequency of positive results in Ibizan hounds [26]. However, both measures of LST reaction intensity, skin thickness and lesion diameter, were significantly higher in Ibizan hounds.

In contrast, statistical analysis failed to find a significant difference between the two breed groups when rSP03B ELISA or RT were considered. However, results suggest a trend for a greater proportion of positive results for RT in Ibizan hounds. Notably, age was considered a significant factor in the results of both tests with rSP03B, and a significant moderate, positive correlation was observed between age (months) and rSP03B EU (*r*_*s*_ = 0.5212). A significant positive relationship was also found for age and SGH EU, albeit to a lesser extent (*r*_*s*_ = 0.2362). This observation coincides with previous studies: older dogs possess greater cumulative exposure from experiencing multiple sand fly seasons [53], which can “prime” dogs for greater anti-sand fly saliva activity even at the beginning of the transmission season, when lower titers would be expected [33]. Because the Ibizan hound group consisted of a lower percentage of adult dogs (< 18 months) with a lower median age than the other breed group, age is thus a likely confounding variable in the rSP03B tests’ data. Considering the GLM analyses, age was determined to be significantly tied to outcome (EU and/or result) of two of the three tests, supporting this possibility.

Interestingly, none of the other markers of a strong cellular immune response alone (i.e. papule presence, IFN-γ production or positive LST) were considered significant factors in anti-*P. perniciosus* antigenic response. Inclusion of the single non-Ibizan hound categorized as papular was thought to influence these unexpected results, as cytological analysis suggested it could be congruent with a different, eosinophilic arthropod bite reaction. Re-classification of the animal as non-papular or its removal from the sample entirely appeared to shift the trend in favor of a stronger anti-*P. perniciosus* antigenic response in dogs with papular dermatitis; however, no trend was found to be statistically significant according to any of the three tests. An important factor to consider is the relationship between papular dermatitis and age, the latter of which is likely a confounding variable in this study, as discussed above. Papular dermatitis is more frequently diagnosed in younger dogs, and the characteristic papules are believed to be the site of a recent sand fly bite and inoculation with *Leishmania* parasites [5, 6], similar to what is observed in the Old World form of human local cutaneous leishmaniasis [54]. As such, dogs presenting with papular dermatitis, largely Ibizan hounds, would be expected to have inherently lower anti-*P. perniciosus* antibody levels than their older, non-papular counterparts.

*Leishmania infantum* seropositivity appeared to be linked to a stronger anti-*P. perniciosus* reaction. Dogs receiving a positive result for *L. infantum* ELISA exhibited a trend for greater EU with either salivary antigen, although the difference was found significant only for rSP03B. Seropositive dogs also were more likely to be considered positive for anti-*P. perniciosus* antibodies according to all three methods, reaching significance in the case of SGH ELISA. GLM results appear to support this relationship: *L. infantum* seropositivity was shown to be a significant factor in four of five of the models. Notably, the seropositive dogs in this study were primarily classified as low- to medium-positive with relatively mild disease, as is common in endemic areas [3]. However, the study included no cases with high sera anti-*Leishmania* antibodies, which are associated with increased disease severity [47]. In order to more thoroughly examine the possible relationship between the anti-sand fly saliva antibodies, anti-*Leishmania* antibodies, and leishmaniosis prognosis, in future studies it would be ideal to include dogs with different levels of specific antibodies against *L*. *infantum* and disease severity.

Conflicting conclusions regarding the relationship between canine antibody response to sand fly saliva and risk of *Leishmania* infection can be found in the literature: studies find either a positive correlation [33–55], a negative correlation [29] or no correlation [33] between anti-*P. perniciosus* saliva and anti-*Leishmania* antibodies. A complicating factor is the variation in anti-*Leishmania* antibodies during an individual dog’s course of infection. Anti-*Leishmania* antibodies are reported to reach a sustained plateau after an initial increase in sick dogs, compared with frequent fluctuations or seroconversion in resistant dogs [56]. Additionally, a positive *L. infantum* ELISA result is not necessarily indicative of *L. infantum* infection, as cross-reaction can occur with other species of *Leishmania* and other parasites, most notably *Trypanosoma* species [57, 58]. However, such cross-reaction is unlikely to occur in the European Mediterranean, where infection with such cases occur less frequently than other endemic areas of CanL. Notably, this study employed additional diagnostic techniques aside from serological detection of *Leishmania* to confirm presence of infection in some dogs.

Herein, the diagnostic performance of three different serological tests detecting *P. perniciosus* exposure was evaluated and compared: traditional SGH ELISA; recombinant protein rSP03B ELISA [34]; and the recently developed rSP03B rapid sero-strip test [38]. SGH ELISA is considered the gold standard, with a higher sensitivity and specificity than either rSP03B ELISA or RT [38]. As in earlier studies in southern Europe [33, 34, 38, 55], a high percentage of agreement (75.76%) was observed between SGH and rSP03B ELISA results along with a significant positive correlation between EU for the two tests (*r*_*s*_ = 0.4759, *df* = 90, *P *<  0.0001). The Cohen’s kappa values comparing SGH ELISA to rSP03B ELISA or RT (0.3846 and 0.3736, respectively) were slightly lower than the values observed (0.67 and 0.83, respectively) in a previous study [38]. Furthermore, the present study found a statistically significant difference in the proportion of positive results between rSP03B ELISA and either SGH ELISA or RT. In the case of rSP03B ELISA, this discrepancy could be attributed to the inherent lower sensitivity of the test, which was compared to SGH ELISA in dogs experimentally exposed to *P. perniciosus* bites [38]. The discrepancy between the rSP03B ELISA and RT likely derives from their respective methods of production; the recombinant SP03B protein for the ELISA was expressed in bacterial cells, whereas the RT protein was purified from Human Embryonic Kidney (HEK) cells [59]. Future studies should include rSP03B proteins produced in HEK cells for both tests to enable a more direct comparison and to avoid undesirable non-specific reactions from co-purified bacterial proteins [38]. Compared to rSP03B, SGH is expected to be more susceptible to cross-reactivity with saliva components from related non-vector or vector sand fly species [31, 60]. Although *P. perniciosus* is one of the most frequently isolated and well-distributed sand fly species on Mallorca, because this study was conducted with naturally infected dogs in an area with multiple species of sand flies [61], such cross-reactivity could very well have influenced these results.

The results of this study are likely influenced by seasonal fluctuations in anti-*P. perniciosus* saliva antibodies. Kinetics of anti-SGH and anti-rSP03B antibodies in dogs are known to mirror patterns in general sand fly activity, peaking during the warmer summer months, when vector density is highest, and dropping substantially during the colder months [33, 55]. The precise start, end, and shape of sand fly density patterns varies substantially between sites based on numerous variables, including latitude, elevation, annual temperature, activity season temperature, and individual year [62]. This variation in vector density, and thus in anti-sand fly saliva antigenic response, could prove challenging for cross-study comparison. In this study, all serum samples were taken during the month of December, in their respective years, which is a period of sand fly inactivity that would be expected to yield lower levels of anti-*P. perniciosus* salivary antibodies. Comparable studies evaluate the anti-sand fly saliva antibody response at the beginning and end of their respective areas’ seasons, although the precise time and total number of samplings vary [29, 33, 34, 55]. This seasonal fluctuation also complicates the comparison of results between salivary antigen tests. A field study using SGH ELISA and RT to evaluate sand fly exposure concluded that in periods of relatively low seroprevalence, such as during the winter, the weak background signal apparent on the RT impedes evaluation of the true degree of agreement between the tests [59]. Indeed, this study observed the majority of dogs clustered about the cut-off for both ELISA tests, with 75% of dogs producing no or faint signal (score of 0 or 1) on the RT.

Ultimately, any conclusions must take into account the small sample size, which could skew results. Expanding the study with an effort to control dog age is recommended. In addition, not all clinical parameters were assessed in each dog due to lack of sufficient sample or clinical justification. Overall, 47 dogs (51.09%) were missing results for at least one of the four main diagnostic tests for *L. infantum* infection. Obtaining a more complete clinical picture of each dog would allow a more thorough examination of the relationship between these and other clinical parameters and the anti-sand fly saliva response. Evaluating other indicators of immune response in peripheral blood could be another possibility; increased expression of the inflammatory cytokines IL-2 and TNF-α is associated with lower disease severity [10], while the chemokine (C-X-C motif) ligand 1 (CXCL1) and chemokine (C-C motif) ligand 2 (CCL2) levels increase with greater disease severity [63]. Upregulation of toll-like receptor 2 (TLR2), the most commonly studied TLR in immunopathogenesis of canine leishmaniosis, has been revealed to correlated with disease progression [11, 19]. Moreover, including more data on parasite load, which was not examined in all dogs, would enable more direct comparison with similar studies with the *L. infantum* vector *L. longipalpis* that consider such parameters [63]. Detecting parasite load in the skin in addition to peripheral blood is also recommended; tissue parasite load was found to closely mirror clinical development of CanL over time and to more accurately reflect host infectivity than parasite load evaluated in bone marrow biopsies [64].

## Conclusions

When compared to other breeds living under similar environmental conditions, Ibizan hounds appear to possess higher levels of anti-*P. perniciosus* saliva antibodies, implying a higher frequency of exposure to sand fly bites. This robust response could be implicated in the stronger parasite-specific, T-cell mediated immune response observed in Ibizan hounds in this study, possibly contributing to the breed’s relative resistance to CanL. The anti-*P. perniciosus* antigenic response also was found to be stronger in older and *L. infantum-*seropositive dogs. Moreover, these findings are observed to a variable extent in multiple measures, including traditional SGH ELISA as well as the less resource- and labor- intensive rSP03B ELISA and rapid sero-strip test. Although further investigation is necessary to confirm these findings, anti-*P. perniciosus* saliva antibodies appear to negatively correlate with susceptibility to *L. infantum* infection, and they show promise as an indicator of CanL prognosis.

## Supplementary information

**Additional file 1: Figure S1.** Example spectrum of rSP03B RT results. RT strips from a positive control and three experimental samples representing the spectrum of RT results. **a** positive control, equivalent to a score of 4. **b** A clear positive band with lower intensity than the control band, classified as 3. **c** A weaker but still positive score of two. **d** A faint band, categorized as one. **e** A negative result, scored as zero. *Notes*: marked with an asterisk, the first band on the nitrocellulose membrane is the test line, the intensity of which is directly related to the amount of anti-rSP03B antibodies present in the sample. The second band is the control line to which the colloidal gold control conjugate binds, indicating that migration has occurred properly and that the test strip is functional.

**Additional file 2: Figure S2.** Papular dermatitis in the inner aspect of the pinnae of two Ibizan hounds clinically suggestive of *L. infantum* infection. Erythematous and slightly scaling papules (**a**) are indicative of acute or early-stage lesions, whereas ulcerated and/or crusted papules with the characteristic “volcanic” appearance represent later-stage lesions (**b**)

## Data Availability

Data supporting the conclusions of this article are provided within the article and its additional files. The datasets used and/or analyzed during the present study are available from the corresponding author upon reasonable request.

## Abbreviations

AIC: Akaike’s information criterion
AUC: area under the curve
CanL: canine leishmaniosis
CCL2: chemokine (C-C motif) ligand 2
CXCL1: chemokine (C-X-C motif) ligand 1
DNA: deoxyribonucleic acid
DTH: delayed type hypersensitivity
ELISA: enzyme-linked immunosorbent assay
EU: ELISA units
GLM: general linear model
IFN-γ: interferon gamma
IL: interleukin
IQR: interquartile range
Ig: immunoglobulin
κ: Cohen’s kappa value
LSA: *L. infantum* soluble antigen
LST: leishmanin skin test
MHC: major histocompatibility complex
NO: nitric oxide
OD: optical density (absorbance)
SE: standard error
SOD: standardized optical density (ELISA units)
PCR: polymerase chain reaction
qPCR: quantitative PCR (real-time PCR)
ROC curve: receiver operating characteristic curve
*r*_*s*_: Spearman rho coefficient
rSP03B RT: rSP03B rapid sero-strip test
SGH: salivary gland homogenate
Th1: type 1 T helper cell (CD4+)
Th2: type 2 T helper cell (CD4+)
TNF-α: tumor necrosis factor alpha
UK: United Kingdom
