# Supplementary figures and images for: Exploring the relationship between susceptibility to canine leishmaniosis and anti-Phlebotomus perniciosus saliva antibodies in Ibizan hounds and dogs of other breeds in Mallorca, Spain
Source: Parasit Vectors. 2020 Apr 21;13:129. doi: 10.1186/s13071-020-3992-8 (PMC7171783; doi:10.1186/s13071-020-3992-8)

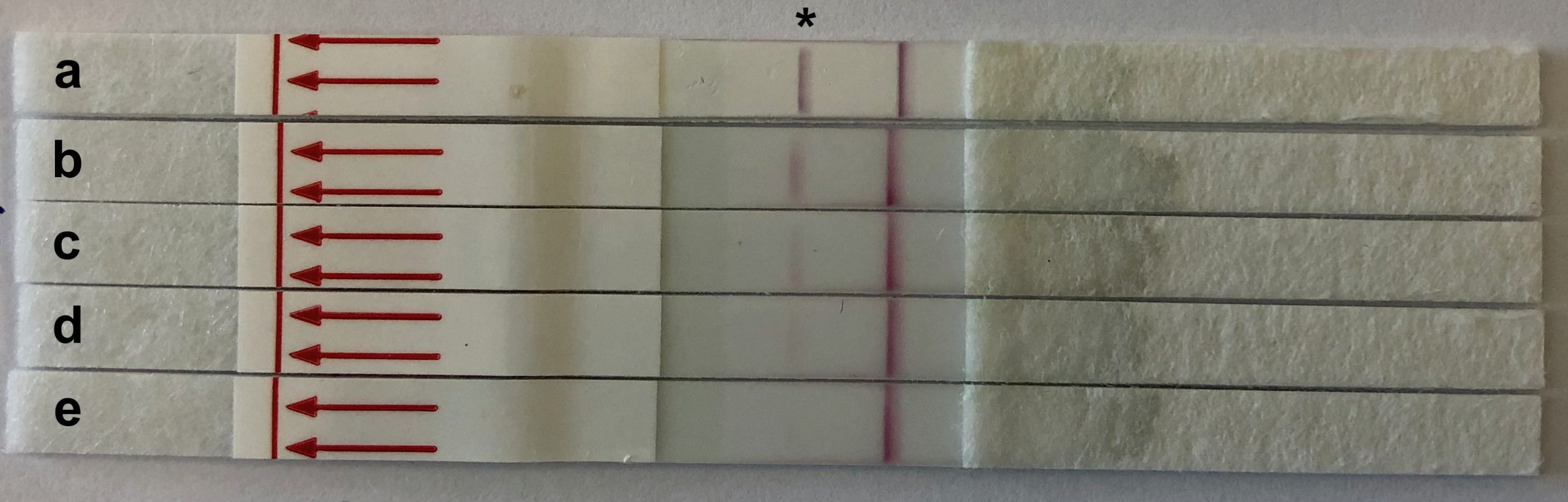

Supplement: Supplementary file 1 — Additional file 1: Figure S1. Example spectrum of rSP03B RT results. RT strips from a positive control and three experimental samples representing the spectrum of RT results. a positive control, equivalent to a score of 4. b A clear positive band with lower intensity than the control band, classified as 3. c A weaker but still positive score of two. d A faint band, categorized as one. e A negative result, scored as zero. Notes: marked with an asterisk, the first band on the nitrocellulose membrane is the test line, the intensity of which is directly related to the amount of anti-rSP03B antibodies present in the sample. The second band is the control line to which the colloidal gold control conjugate binds, indicating that migration has occurred properly and that the test strip is functional. [file 13071_2020_3992_MOESM1_ESM.tif]

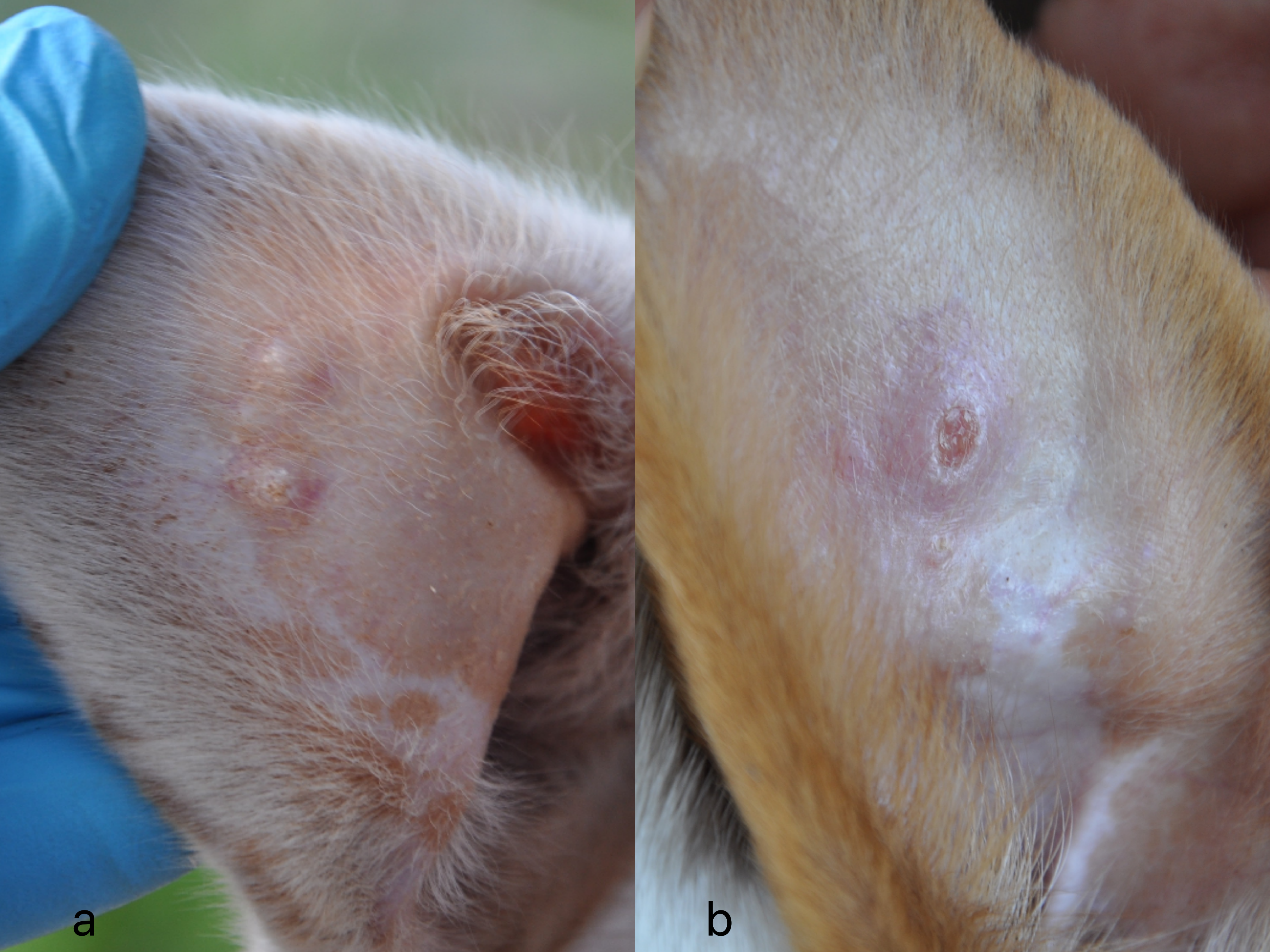

Supplement: Supplementary file 2 — Additional file 2: Figure S2. Papular dermatitis in the inner aspect of the pinnae of two Ibizan hounds clinically suggestive of L. infantum infection. Erythematous and slightly scaling papules (a) are indicative of acute or early-stage lesions, whereas ulcerated and/or crusted papules with the characteristic “volcanic” appearance represent later-stage lesions (b) [file 13071_2020_3992_MOESM2_ESM.tiff]
